# Safety and efficacy of panitumumab in combination with trifluridine/tipiracil for pre-treated patients with unresectable, metastatic colorectal cancer with wild-type *RAS*: The phase 1/2 APOLLON study

**DOI:** 10.1007/s10147-021-01902-2

**Published:** 2021-04-29

**Authors:** Takeshi Kato, Yoshinori Kagawa, Yasutoshi Kuboki, Makio Gamoh, Yoshito Komatsu, Hirofumi Yasui, Hironaga Satake, Eiji Oki, Hiroaki Tanioka, Masahito Kotaka, Akitaka Makiyama, Tadamichi Denda, Masahiro Goto, Takayuki Yoshino, Kentaro Yamazaki, Junpei Soeda, Kazunori Shibuya, Masaru Iwata, Koji Oba, Kensei Yamaguchi

**Affiliations:** 1 National Hospital Organization Osaka National Hospital, 2 Chome-1-14 Hoenzaka, Chuo Ward, Osaka, 540-0006 Japan; 2 Kansai Rosai Hospital, 3 Chome-1-69 Inabaso, Amagasaki, Hyogo 660-8511 Japan; 3 National Cancer Center Hospital East, 6 Chome-5-1 Kashiwanoha, Kashiwa, Chiba 277-8577 Japan; 4 Osaki Citizen Hospital, Furukawa Honami, 3 Chome, Osaki, 989-6183 Japan; 5 Hokkaido University Hospital, 5 Chome Kita 14 Jonishi, Kita Ward, Sapporo, Hokkaido 060-8648 Japan; 6 Shizuoka Cancer Center, 1007 Shimonagakubo, Nagaizumi, Sunto District, Shizuoka, 411-0934 Japan; 7 Kobe City Medical Center General Hospital, 2 Chome-1-1 Minatojima Minamimachi, Chuo Ward, Kobe, Hyogo 650-0047 Japan; 8 Kyushu University, Maidashi 3 Chome-1-3, Higashi Ward, Fukuoka, 812-0053 Japan; 9 Okayama Rosai Hospital, 1 Chome-10-25 Chikkomidorimachi, Minami Ward, Okayama, 702-8055 Japan; 10 Sano Hospital, 2 Chome-5-1 Shimizugaoka, Tarumi Ward, Kobe, Hyogo 655-0031 Japan; 11 Japan Community Healthcare Organization Kyushu Hospital, 1 Chome-8-1 Kishinoura, Yahatanishi Ward, Kitakyushu, Fukuoka 806-8501 Japan; 12 Gifu University Hospital, 1-1 Yanagido, Gifu, 501-1194 Japan; 13 Chiba Cancer Center, 666-2 Nitona-cho, Chuo Ward, Chiba, 260-8717 Japan; 14 Osaka Medical College Hospital, 2-7 Daigakumachi, Takatsuki, Osaka 569-0096 Japan; 15 Takeda Pharmaceutical Company, Ltd, Nihonbashi-Honcho 2 Chome-1-1, Chuo Ward, Tokyo, 103-8668 Japan; 16 University of Tokyo, 7 Chome-3-1 Hongo, Bunkyo, Tokyo, 113-8654 Japan; 17 Gastroenterological Chemotherapy Department, Cancer Institute Hospital of Japanese Foundation for Cancer Research, 3 Chome-8-31, Ariake, Koto, Tokyo, 135-8550 Japan; 18 Present Address: Cancer Treatment Center, Kansai Medical University Hospital, 2 Chome-3-1 Shinmachi, Hirakata, Osaka 573-1191 Japan; 19 Present Address: Medical Oncology Kawasaki Medical School, 577 Matsushima, Kurashiki, Okayama 701-0192 Japan

**Keywords:** Salvage line, Anti-EGFR antibody, Phase 1/2 clinical trial, Oral nucleoside anti-tumour agent, Outcomes

## Abstract

**Background:**

We aimed to assess the safety and efficacy of combination treatment with panitumumab plus trifluridine/tipiracil (FTD/TPI) in patients with wild-type RAS metastatic colorectal cancer (mCRC) who were refractory/intolerant to standard therapies other than anti-epidermal growth factor receptor therapy.

**Methods:**

APOLLON was an open-label, multicentre, phase 1/2 trial. In the phase 1 part, 3 + 3 de-escalation design was used to investigate the recommended phase 2 dose (RP2D); all patients in the phase 2 part received the RP2D. The primary endpoint was investigator-assessed progression-free survival (PFS) rate at 6 months. Secondary endpoints included PFS, overall survival (OS), overall response rate (ORR), disease control rate (DCR), time to treatment failure (TTF), and safety.

**Results:**

Fifty-six patients were enrolled (phase 1, *n* = 7; phase 2, *n* = 49) at 25 Japanese centres. No dose-limiting toxicities were observed in patients receiving panitumumab (6 mg/kg every 2 weeks) plus FTD/TPI (35 mg/m^2^ twice daily; days 1–5 and 8–12 in a 28-day cycle), which became RP2D. PFS rate at 6 months was 33.3% (90% confidence interval [CI] 22.8–45.3). Median PFS, OS, ORR, DCR, and TTF were 5.8 months (95% CI 4.5–6.5), 14.1 months (95% CI 12.2–19.3), 37.0% (95% CI 24.3–51.3), 81.5% (95% CI 68.6–90.8), and 5.8 months (95% CI 4.29–6.21), respectively. Neutrophil count decreased (47.3%) was the most common Grade 3/4 treatment-emergent adverse event. No treatment-related deaths occurred.

**Conclusion:**

Panitumumab plus FTD/TPI exhibited favourable anti-tumour activity with a manageable safety profile and may be a therapeutic option for pre-treated mCRC patients.

**Supplementary Information:**

The online version contains supplementary material available at 10.1007/s10147-021-01902-2.

## Introduction

Colorectal cancer (CRC) is the third most common cancer worldwide, and the second most common cause of cancer mortality [1]. Unresectable metastatic CRC (mCRC) is essentially incurable and the prognosis remains poor, with 5-year overall survival (OS) < 20% [2]. Recently, treatment advances in the first- and second-line settings, including the use of combination therapies, such as doublet and triplet chemotherapy (e.g. 5-fluorouracil, irinotecan, and oxaliplatin), alongside targeted biological agents directed against vascular endothelial growth factor (VEGF) and epidermal growth factor receptor (EGFR), have resulted in improvements in OS [3]. Nevertheless, more effective drugs and/or regimens are needed to improve survival and the control of tumour progression [4].

The oral nucleoside anti-tumour agent trifluridine/tipiracil (FTD/TPI, known as TAS-102), consisting of trifluridine, a thymidine-cased nucleic acid analogue, and tipiracil hydrochloride, showed a significant OS benefit with an acceptable safety profile in patients with refractory mCRC [5, 6]. The anti-EGFR monoclonal antibody panitumumab was effective in patients with mCRC, both when administered as monotherapy and in combination with chemotherapy [3, 7–9]. Patients with wild-type *RAS* tumours (i.e. wild-type for *KRAS* and *NRAS*, exons 2, 3, and 4) by extended *RAS* analysis presented numerically improved survival in the first- [10], second- [9], and third-line [11] settings.

In preclinical studies, panitumumab suppressed FTD-induced EGFR-mediated responses, such as ERK/AKT/STAT3 activation, in vitro [12]. Panitumumab plus FTD/TPI had a greater activity leading to tumour regression against CRC xenografts than either drug alone in vivo [12]. Thus, combining FTD/TPI with panitumumab could be beneficial; however, published data reporting the safety and efficacy of an anti-EGFR agent plus FTD/TPI for mCRC are lacking. We assessed the safety and efficacy of combination treatment with panitumumab plus FTD/TPI in patients with wild-type *RAS* mCRC who were refractory or intolerant to standard therapies other than anti-EGFR therapy.

## Materials and methods

### Study design and participants

APOLLON was an open-label, single-arm, multicentre, phase 1/2 trial designed to assess the combination of panitumumab plus FTD/TPI at 25 Japanese centres. The phase 1 portion determined the recommended phase 2 dose (RP2D) in a 3 + 3 dose de-escalation design at four centres. In phase 2, all patients received the RP2D.

The study conduct was in accordance with the Declaration of Helsinki, the International Conference on Harmonisation for Good Clinical Practice guidelines, and Ethical Guidelines for Clinical Research in Japan. The institutional review boards approved the protocol. All patients provided written informed consent. The study is registered with the university hospital Medical Information Network Clinical Trials Registry (number UMIN000019876).

Major inclusion criteria were patients aged 20–74 years; histologically confirmed unresectable, metastatic colorectal adenocarcinoma; refractory or intolerant to fluoropyrimidines, irinotecan, oxaliplatin, and angiogenesis inhibitors (e.g. bevacizumab, ziv-aflibercept, or ramucirumab); measurable lesions based on the Response Evaluation Criteria in Solid Tumours (RECIST version 1.1); Eastern Cooperative Oncology Group performance status (ECOG PS) of 0 or 1; and presence of wild-type *KRAS/NRAS*. Major exclusion criteria were history of treatment with any anti-EGFR drugs (cetuximab or panitumumab), regorafenib, or FTD/TPI and presence of known v-raf murine sarcoma viral oncogene homolog B1 (*BRAF*) mutation.

### Procedures

In phase 1, the starting dose was FTD/TPI 35 mg/m^2^ given orally twice daily on days 1–5 and 8–12 in a 28-day cycle, plus panitumumab (6 mg/kg) administered by intravenous (IV) infusion for 60 min every 2 weeks (Q2W) in a 28-day cycle until tumour progression, unacceptable toxic effects, or withdrawal of consent. The RP2D was determined using a standard 3 + 3 format.

Dose-limiting toxicities (DLTs) were defined as Grade ≥ 3 non-haematological toxicities, excluding controllable nausea, vomiting, and transient electrolyte abnormalities; Grade 4 neutrophil count decreased lasting > 7 days; Grade ≥ 3 febrile neutropenia; Grade 4 thrombocytopenia; or unresolved toxicities leading to a > 2-week delay until the next cycle. Toxicities were graded according to the CTCAE version 4.03.

In phase 2, all patients received the RP2D, but dose modifications were allowed for safety in patients who developed treatment-emergent adverse events (TEAEs). Patients who required longer than a 28-day dose interruption for adverse events (AEs) were withdrawn from the study. If patients had unacceptable toxicities related to panitumumab, treatment with FTD/TPI monotherapy could be permitted. If the start of a treatment cycle was delayed, panitumumab administration could be delayed until the next cycle of FTD/TPI was administered. Additional details of the study methods are provided in Online Resource 1.

### Outcomes

The primary endpoint during the phase 2 portion was investigator-assessed progression-free survival (PFS) rate at 6 months. Enhanced computed tomography or magnetic resonance imaging of the chest, abdomen, and pelvis were performed every 8 weeks during the first year after treatment initiation and every 12 weeks thereafter and assessed according to RECIST 1.1. The absence of progression at 6 months was defined as the proportion of patients who survived without disease progression at 24 ± 2 weeks, per investigator assessment, based on imaging data. If no data were available at this time point, patients were classified as not having achieved PFS at 6 months. An independent central review was not conducted for PFS evaluation.

Secondary endpoints were investigator-assessed PFS, OS, overall response rate (ORR), disease control rate (DCR), time to treatment failure (TTF), and safety. TEAEs were assessed every 2 weeks and coded using the Medical Dictionary for Regulatory Activities version 21.0.

### Statistical analysis

For phase 1, a safety review committee reviewed safety data and confirmed dose-escalation decisions. No formal power calculations were done. Safety data were summarised descriptively. For phase 2, we set 48% as a promising result and 29% as a poor result, based on previous panitumumab data from the phase 3 ASPECCT study [13], with a one-sided α of 5% and power of 90%. A single-stage binomial design required 46 patients, including phase 1 patients who received the RP2D.

The full analysis set (FAS), comprising patients who received at least one dose of FTD/TPI and panitumumab at the RP2D, was used for efficacy analyses. Safety analyses included all enrolled patients who received at least one dose of study medication at any dosage. For the primary endpoint during the phase 2 portion, the proportion of patients with PFS at 6 months was calculated with exact binomial 90% confidence intervals (CIs) using the Clopper-Pearson method. For overall PFS, TTF, duration of response, and OS, survival curves and median values were generated using the Kaplan–Meier method with 95% CIs calculated by use of Greenwood’s formula. Subgroup analyses for OS, PFS, and ORR were undertaken, focusing on pre-defined baseline patient factors, including age, sex, ECOG PS, and primary tumour site, and the post hoc factors of chemotherapy-induced neutropenia (CIN) within 6 weeks and early tumour shrinkage (ETS) within 8 weeks. Adjusted hazard ratios and their corresponding 95% CIs were estimated using the multiple Cox proportional hazards model for time-to-event outcome. Adjusted odds ratios and their corresponding 95% CIs were estimated using a multiple logistic regression model for binary outcomes. All analyses were performed using SAS version 9.4 (SAS Institute, NC, USA).

## Results

**Fig. 1:**
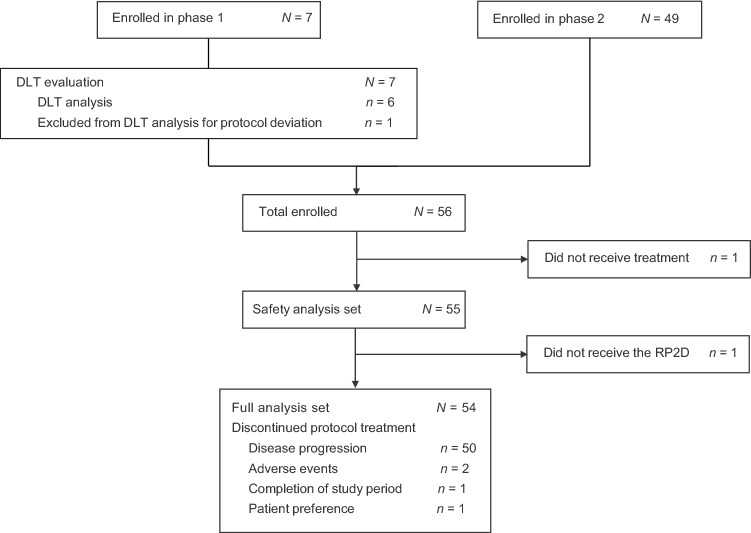
Trial profile. DLT = dose-limiting toxicity; RP2D = recommended phase 2 dose

Between December 8, 2015, and April 5, 2017, 56 patients with mCRC were enrolled in the study: seven in phase 1 and 49 in phase 2 (Fig. 1). In phase 1, one patient received granulocyte-colony stimulating factor (for neutrophil count decreased [Grade 3] without fever), which was classified as a protocol deviation for this phase and was therefore excluded from the DLT analysis. No DLT was observed in the remaining six DLT-evaluable patients who received the initial dose of FTD/TPI (35 mg/m^2^ twice a day orally on days 1–5 and 8–12 in a 28-day cycle) plus panitumumab (6 mg/kg, 60-min IV Q2W), which was subsequently regarded as the RP2D. In phase 2, all seven patients who received phase 1 treatment plus an additional 49 patients were enrolled. Of these 56 patients, 55 formed the safety analysis set; one patient who did not receive any study treatment was excluded owing to worsening of PS. One additional patient was excluded from the FAS (*n* = 54) due to an infusion reaction during the first panitumumab treatment and was not able to receive study treatment at the RP2D. Thus, a total of two patients were excluded from the FAS.

Baseline characteristics of the safety analysis set are summarised in Table 1. Over half of patients (29/55 [52.7%]) were male, with a median age of 63 years. The majority of patients (38/55 [69.1%]) had an ECOG PS of 0, and most (49/55 [89.1%]) had received two or more prior chemotherapy regimens.

**Table 1:** Baseline characteristics

	All patients (*N* = 55)
Age, years	63 (38–74)
Sex	
Male	29 (52.7%)
Female	26 (47.3%)
ECOG performance status	
0	38 (69.1%)
1	17 (30.9%)
Primary site^a^	
Right-sided colon	7 (12.7%)
Left-sided colorectal	48 (87.3%)
Number of metastatic sites	
1	23 (41.8%)
≥ 2	32 (58.2%)
Metastatic sites	
Liver	33 (60.0%)
Lung	28 (50.9%)
Peritoneum	4 (7.3%)
Lymph nodes	26 (48.3%)
Bone	4 (7.3%)
Adrenal gland	3 (5.5%)
Others	3 (5.5%)
Resection of primary tumour	
Yes	43 (78.2%)
No	12 (21.8%)
Adjuvant chemotherapy	
Yes	16 (29.1%)
No	39 (70.9%)
Number of prior chemotherapies	
1	6 (10.9%)
2	37 (67.3%)
≥ 3	12 (21.8%)
Duration of prior chemotherapy, days	595.5 (72–2240)

Data are *n* (%), or median (range). Primary tumour location and metastatic organ could be counted more than once

*ECOG* Eastern Cooperative Oncology Group

^a^Right-sided colon cancer was defined as cancer located in the cecum, ascending colon, or transverse colon, and left-sided colorectal cancer was defined as cancer in the descending colon, sigmoid colon, rectosigmoid colon, or rectum

After a median follow-up of 16.5 months (data cut-off March 30, 2018), median relative dose intensities (RDIs) of panitumumab and FTD/TPI were 73.7% (range 36.8–100.0) and 73.8% (range 34.5–98.8), respectively (observation periods; 6 months from start of study treatment). Treatment administration status is shown in Online Resource 2.

**Fig. 2:**
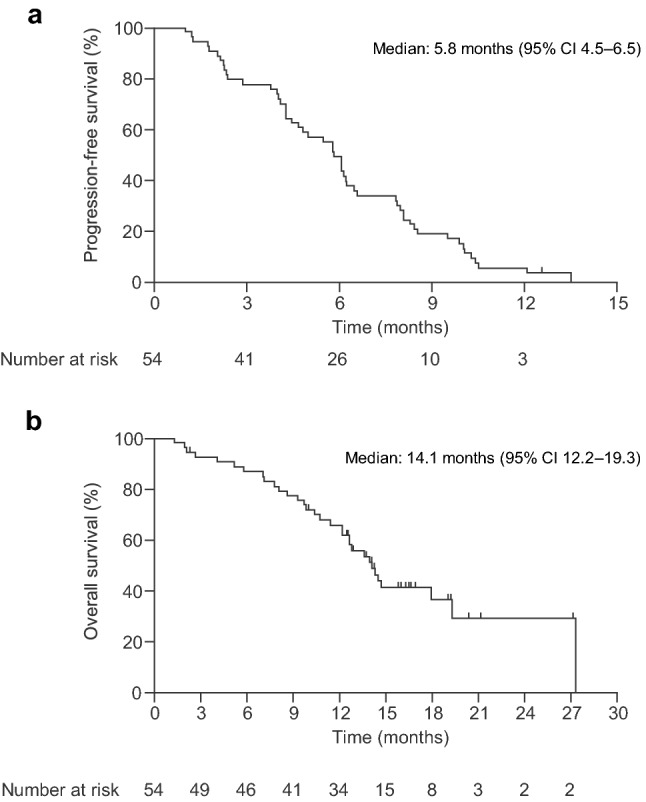
Kaplan–Meier curves of **a** investigator-assessed progression-free survival and **b** overall survival in the full analysis set. CI = confidence interval

In the FAS, the PFS rate at 6 months was 33.3% (90% CI 22.77%–45.32%; *p* = 0.2414). The null hypothesis (i.e. PFS rate ≤ 29%) was not rejected; thus, the primary endpoint was not met. Median PFS and OS were 5.8 months (95% CI 4.5–6.5) and 14.1 months (95% CI 12.2–19.3), respectively (Fig. 2, Online Resource 2). The ORR and DCR were 37.0% (95% CI 24.3–51.3) and 81.5% (95% CI 68.6–90.8), respectively (Table 2, Online Resource 2). The TTF was 5.8 months (95% CI 4.29–6.21).

**Table 2:** Best response to treatment

Objective response, *n* (%, 95% CI)	Full analysis set (*n* = 54)
Complete response	0
Partial response	20 (37.0%)
Stable disease	24 (44.4%)
Progressive disease	11 (18.5%)
Objective response rate	20 (37.0%, 24.3–51.26)
Disease control rate	44 (81.5%, 68.6–90.8)

Data are *n* (%) or *n* (%, 95% CI)

*CI* confidence interval

Regarding TEAEs, 42/55 patients (76.4%) experienced at least one Grade 3/4 TEAE, the most common of which included neutrophil count decreased (Grade 3, 30.9%; Grade 4, 16.4%), febrile neutropenia (Grade 3, 10.9%), stomatitis (Grade 3, 9.1%), dermatitis acneiform (Grade 3, 9.1%), fatigue (Grade 3, 3.6%), and hypomagnesemia (Grade 3, 3.6%) (Table 3). No unexpected or treatment-related deaths occurred.

**Table 3:** Treatment-emergent adverse events during study treatment period (safety analysis set)

Adverse event	All grades	Grade 3	Grade 4
Haematological			
Neutrophil count decreased	36 (65.5%)	17 (30.9%)	9 (16.4%)
Platelet count decreased	13 (23.6%)	0	0
Anaemia	9 (16.4%)	2 (3.6%)	0
White blood cell count decreased	7 (12.7%)	1 (1.8%)	1 (1.8%)
Non-haematological			
Stomatitis	39 (70.9%)	5 (9.1%)	0
Dermatitis acneiform	34 (61.8%)	5 (9.1%)	0
Decreased appetite	30 (54.5%)	1 (1.8%)	0
Fatigue	27 (49.1%)	2 (3.6%)	0
Dry skin	27 (49.1%)	2 (3.6%)	0
Nausea	23 (41.8%)	1 (1.8%)	0
Paronychia	23 (41.8%)	0	0
Diarrhoea	17 (30.9%)	1 (1.8%)	0
Palmar-plantar erythrodysaesthesia syndrome	14 (25.5%)	1 (1.8%)	0
Platelet count decreased	13 (23.6%)	0	0
Hypomagnesaemia	12 (21.8%)	2 (3.6%)	0
Dysgeusia	12 (21.8%)	0	0
Peripheral sensory neuropathy	11 (20.0%)	2 (3.6%)	0
Rash	11 (20.0%)	1 (1.8%)	0
Malaise	10 (18.2%)	0	0
Vomiting	9 (16.4%)	1 (1.8%)	0
Pyrexia	9 (16.4%)	0	0
Pruritus	9 (16.4%)	0	0
Febrile neutropenia	6 (10.9%)	6 (10.9%)	0
Abdominal pain	6 (10.9%)	0	0

Data are *n* (%). The safety population includes all enrolled patients who received at least one dose of study treatment (*n* = 55). No treatment-related deaths occurred

Subsequent treatments after the discontinuation of study treatment are shown in the appendix (Online Resource 3). After study treatment discontinuation, 39/55 patients received subsequent treatment; most of these (25/39) were treated with regorafenib.

**Fig. 3:**
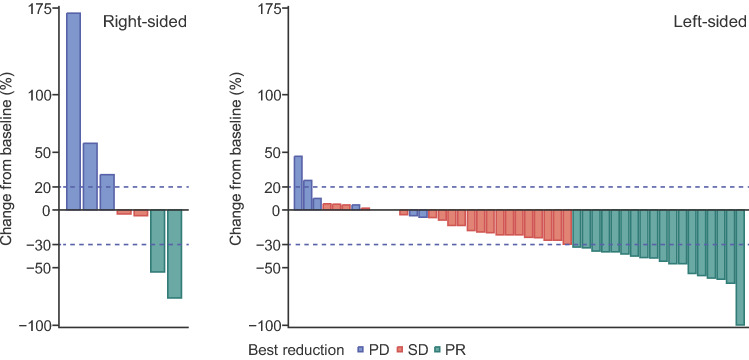
Best response in patients treated with the recommended phase 2 dose of trifluridine/tipiracil plus panitumumab according to primary tumour location. PD = progressive disease; PR = partial response; SD = stable disease

Ad hoc analysis for pre-defined baseline patient factors indicated no prognostic factors for PFS, OS, or ORR (Online Resource 4–6). In the analysis using post hoc factors, ETS (20% tumour reduction within 8 ± 1 weeks of initiation of study treatment) occurred in 19/54 patients. Compared with patients without ETS, those with ETS had a significantly prolonged OS; however, significant increases in PFS were not seen (Online Resource 7 and 8). Patients with left-sided tumours had relatively better PFS and OS compared with those with right-sided tumours (Online Resource 9); however, partial response and a relatively long duration of response were observed in 2/7 patients with right-sided tumours (Fig. 3, Online Resource 10). In addition, there was no evidence of the initial onset of skin toxicity, neutropenia, or stomatitis having an effect on PFS or OS outcomes.

## Discussion

To the best of our knowledge, this trial is the first prospective phase 1/2 study to evaluate the anti-tumour activity and safety of the combination of panitumumab plus FTD/TPI as salvage therapy in patients with mCRC. In this study, FTD/TPI was safely combined with panitumumab in patients with mCRC and the median PFS and OS reached 5.8 months and 14.1 months, respectively. These survival durations were longer than those previously seen with each drug alone but similar to the combination of panitumumab and irinotecan: for FTD/TPI, median PFS was reported to be 2.0 months and median OS was 7.1 months [5]; for panitumumab, median PFS was 5.2 months and median OS was 10.0 months [11]; and for panitumumab and irinotecan, median PFS was 5.4 months and median OS was 14.9 months [14]. Toxicities associated with the combination regimen were generally mild and well tolerated. Overall, both haematological and non-haematological AEs observed in this study were similar to those reported for each monotherapy. However, the frequency of stomatitis (all grades, 70.9%/Grade ≥ 3, 9.1%) was increased compared with each agent alone (FTD/TPI, 8%/ < 1%; [5] panitumumab, 5.2%/0.6% [13]), possibly due to the overlapping toxicity effects between FTD/TPI and panitumumab. Notably, CIN was increased with this combination compared with FTD/TPI monotherapy. Generally, panitumumab does not enhance neutropenia when used with other cytotoxic drug(s); [7, 15] there are no clear reasons for the enhancement observed in our study.

As the lower limit of the 90% CI of the PFS rate at 6 months did not exceed the pre-specified threshold, our trial did not meet the primary endpoint. One possible reason for this may be that all patients in our study had received prior anti-VEGF agents at enrolment, whereas the threshold value of 29% used for the statistical hypothesis testing was based on the results of the ASPECCT study, in which only 30% of included patients had received prior bevacizumab (a VEGF inhibitor) [13]. Results from previous preclinical [16] and clinical studies [17, 18] have demonstrated that the efficacy of anti-EGFR therapy can differ depending on specific patient background factors, such as prior anti-VEGF treatment; thus, the threshold of 29% used in our study might have been high. Nonetheless, the median PFS and OS data from our study suggest that panitumumab plus FTD/TPI can provide efficacy even in patients pre-treated with anti-VEGF agents, such as bevacizumab; these results are in line with a prior analysis which demonstrated that panitumumab was more efficacious than cetuximab when administered after a bevacizumab-containing regimen [19].

Another reason for the lower-than-expected PFS rate at 6 months may be that the treatment modifications and postponements recorded in this study resulted in the RDIs for both FTD/TPI and panitumumab being reduced to approximately 70%. This was lower than in previous studies of both drugs as monotherapy (85.7% for FTD/TPI [6] and 99% for panitumumab [13]) and may have affected efficacy rates in our study. Although we cannot be certain of the reasons, a high frequency of dose reduction and suspension due to CIN may be one possible cause as many cases of neutrophil count decreased in this study were severe (Grade 3/4). It is also possible that even if patients had Grade ≥ 3 CIN and could have continued panitumumab monotherapy while suspending FTD/TPI due to Grade ≥ 3 CIN, investigators may have decided to simply suspend both treatments; this would account for the low panitumumab RDI. Recently, it was reported that in heavily pre-treated patients (refractory to chemotherapy, anti-VEGF, and anti-EGFR therapy [for tumours with wild-type *RAS*]) biweekly administration of FTD/TPI with bevacizumab showed promising anti-tumour activity with acceptable toxicity and dramatically decreased CIN compared with the standard combination treatment schedule [20]. Thus, if panitumumab was combined with biweekly FTD/TPI, it may be possible to decrease CIN and maintain the RDI of both drugs, although additional studies are required to explore this possibility.

The ORR in our study was relatively high (37%) in patients who were refractory or intolerant to standard therapies. Several monotherapeutic regimens are currently used for standard of care salvage therapy for mCRC [21], and compared with these regimens, the high rate of response with panitumumab plus FTD/TPI was notable. There is an inherent therapeutic limitation to monotherapy, since drugs which inhibit a single oncogenic driver cannot provide the same benefit to all mCRC patients due to heterogeneity in both patient and tumour characteristics. Thus, regimens combining panitumumab with cytotoxic drugs are likely to be more effective than panitumumab monotherapy; indeed, combining panitumumab with chemotherapy was previously reported to have superior efficacy compared with monotherapy [22]. Panitumumab plus FTD/TPI interacts with signalling cascades, such as the mitogen-activated protein kinase/extracellular signal-regulated kinase pathway [12], potentially increasing the cytoreductive efficacy of the combination. A previous report showed that higher response might improve quality of life, including ameliorating pain and anorexia [23], and intensive treatment for rapid reduction in tumour burden is needed for patients with impending clinical threat, organ dysfunction, or severe disease-related symptoms [3]. As such, the rapid reduction of tumour burden by panitumumab plus FTD/TPI could be beneficial in such patients.

In prior studies, patients with left-sided tumours receiving chemotherapy plus an anti-EGFR antibody have demonstrated superior treatment outcomes in terms of PFS, OS, and ORR compared with patients with right-sided tumours receiving the same therapy [4, 9]. Currently, anti-VEGF, rather than anti-EGFR, agents are usually recommended as first-line and subsequent therapy for patients with right-sided primary tumours [24, 25]. An analysis of data from four previous clinical studies in which panitumumab was used as second- and later-line treatment for mCRC, indicated that OS was markedly worse and the response rate was lower in patients with right-sided tumours compared with those with left-sided tumours, particularly when panitumumab was administered as monotherapy [26]. Similar results have also been reported for cetuximab [27]. Nonetheless, 2/7 patients with right-sided tumours in our study recorded partial response and a long duration of response, although a limited number of patients were evaluated. This may indicate that this combination regimen may be effective regardless of primary tumour location and could represent a possible option for salvage therapy after the failure of front-line regimens with anti-VEGF agents.

Previous exploratory studies have shown that early-onset AEs observed after treatment with panitumumab or FTD/TPI, especially skin toxicity and neutrophil count decreased, may be predictive factors for later efficacy outcomes [28, 29]. Obvious predictive factors associated with early onset of AEs on later efficacy outcomes were not observed in our study; however, PFS and OS appeared to be worsened in patients without early onset of both neutrophil count decreased and skin toxicity.

This study had some limitations that must be considered when evaluating the resulting data. This was a non-randomised trial with a small sample size; in particular, there were only seven patients with right-sided mCRC. *BRAF* mutation testing was not performed in all patients prior to enrolment, and only patients with known *BRAF* mutations were excluded. An exploratory biomarker study was not conducted. Additionally, no patients received regorafenib as previous treatment. Panitumumab plus irinotecan combination therapy is one of the standard third-line therapy options for patients with mCRC. However, based on the relatively high ORR of 37%, and compared with the ORR in a previous study on panitumumab plus irinotecan combination therapy (26%) [14], our results suggest that this combination therapy could be a treatment option as salvage therapy for patients with mCRC refractory or intolerant to fluoropyrimidine, irinotecan, oxaliplatin, and anti-angiogenesis therapy.

In conclusion, panitumumab plus FTD/TPI has favourable activity and a manageable safety profile in patients with mCRC who are refractory or intolerant to standard chemotherapy. Further investigations, especially studies comparing this regimen with panitumumab and irinotecan combination therapy, are required to validate the potential activity of this regimen in patients with mCRC. These days, panitumumab is increasingly used as first-line treatment for patients with left-sided tumours and may be used to rechallenge *RAS* wild-type tumours in later-line treatment. Further analyses of such rechallenge strategies [30] will contribute additional information on using an anti-EGFR antibody in combination with FTD/TPI, and studies are now in progress (UMIN000027210).

## Conflict of interest

TK reports personal fees from Takeda during the conduct of the study; and personal fees from Bayer, Lilly, Yakult Honsha, Sanofi; and personal fees and research grants from Chugai Pharma. YKu reports research grants from Takeda during the conduct of the study; and personal fees from Bayer and Lilly; research grants from Incyte, AstraZeneca, and Daiichi Sankyo; and personal fees and research grants from Taiho Pharmaceutical. MG reports personal fees from Yakult Honsha, Ono Pharmaceutical, Lilly Japan, and Merck Serono; research grants from Kyowa Hakko Kirin; and personal fees and research grants from Chugai Pharma, Taiho Pharmaceutical, Nippon Kayaku, and Mochida Pharmaceutical. YKo reports personal fees from Merck and Pfizer; research grants from MSD, Ono Pharmaceutical, and Yakult Honsha; and personal fees and research grants from Taiho Pharmaceutical, Lilly, Novartis, Bayer, and Chugai Pharma. HS reports personal fees from Takeda during the conduct of the study; and personal fees from Bayer, Bristol-Myers Squibb, Chugai Pharma, Daiichi Sankyo, Eli Lilly Japan, Merck Bio Pharma, MSD, Ono Pharmaceutical, Sanofi, Taiho Pharmaceutical, Takeda, and Yakult Honsha. EO reports personal fees from Takeda during the conduct of the study; and personal fees from Taiho Pharmaceutical, Chugai Pharma, Lilly, Merck Serono, Yakult Honsha, Ono Pharmaceutical, and Bayer. HT reports personal fees and research grants from Takeda during the conduct of the study; personal fees from Ono Pharmaceutical, Chugai Pharma, Merck Serono, and Taiho Pharmaceutical; and personal fees and research grants from Taiho Pharmaceutical. MK reports personal fees from Takeda during the conduct of the study; and personal fees from Chugai Pharma, Yakult Honsha, Merck Serono, Taiho Pharmaceutical, and Lilly. AM reports personal fees from Takeda during the conduct of the study; and personal fees from Lilly and Chugai Pharma. TD reports personal fees from Takeda during the conduct of the study; and personal fees from Taiho Pharmaceutical, Yakult Honsha, and Chugai Pharma; and research grants from Sanofi, Boehringer Ingelheim, and MSD. MG reports personal fees from Takeda during the conduct of the study; and personal fees from Taiho Pharmaceutical, Chugai Pharma, Yakult, Ono Pharmaceutical, Daiichi Sankyo, Nippon Kayaku, and Lilly. TY reports research grants from Chugai Pharma, Sanofi, Sumitomo Dainippon, and GlaxoSmithKline plc. KYamaz reports personal fees and research grants from Takeda during the conduct of the study; personal fees from Chugai Pharma, Daiichi Sankyo, Yakult Honsha, Bayer, Merck Serono, Bristol-Myers Squibb Japan, Lilly, Sanofi, and Ono Pharmaceutical; and personal fees and research grants from Taiho Pharmaceutical. JS, KS, and MI report employment with Takeda during the conduct of the study. KO reports personal fees from Eisai, Chugai Pharma, Daiichi Sankyo, and Asahi Kasei. KYamag reports personal fees from Takeda during the conduct of the study; personal fees from Chugai Pharma, Merck Serono, Bristol-Myers Squibb Japan, and Sanofi; research grants from MSD Oncology, Dainippon Sumitomo Pharma, Daiichi Sankyo, and Gilead Sciences; and personal fees and research grants from Ono Pharmaceutical, Taiho Pharmaceutical, Lilly, and Yakult Honsha. All other authors declare that they have no conflict of interest.

## Ethics approval

The study conduct was in accordance with the Declaration of Helsinki, the International Conference on Harmonisation for Good Clinical Practice guidelines, and Ethical Guidelines for Clinical Research in Japan. The institutional review boards approved the protocol. The study is registered with ClinicalTrials.gov (number NCT02613221), the university hospital Medical Information Network Clinical Trials Registry (number UMIN000019876), and Japan Pharmaceutical Information Center Clinical Trials Information (number JapicCTI-153076).

## Supplementary Information

Below is the link to the electronic supplementary material.

Supplementary file 1 (DOCX 254 KB)

## Data Availability

The study protocol and statistical analysis plan are available for download at https://clinicaltrials.gov/ct2/show/NCT02613221. Takeda makes patient-level, de-identified data sets and associated documents available after applicable marketing approvals and commercial availability have been received, an opportunity for the primary publication of the research has been allowed, and other criteria have been met as set forth in Takeda’s Data Sharing Policy (see https://www.takedaclinicaltrials.com for details). To obtain access, researchers must submit a legitimate academic research proposal for adjudication by an independent review panel, who will review the scientific merit of the research and the requestor’s qualifications and conflict of interest that can result in potential bias. Once approved, qualified researchers who sign a data sharing agreement are provided access to these data in a secure research environment.
