# Supplementary material for: Safety and efficacy of panitumumab in combination with trifluridine/tipiracil for pre-treated patients with unresectable, metastatic colorectal cancer with wild-type RAS: The phase 1/2 APOLLON study
Source: Int J Clin Oncol. 2021 Apr 29;26(7):1238–47. doi: 10.1007/s10147-021-01902-2 (PMC8213662; doi:10.1007/s10147-021-01902-2)
Supplement: Supplementary file 1 — (DOCX 254 KB) [file 10147_2021_1902_MOESM1_ESM.docx]

**Online Resources**

# Safety and efficacy of panitumumab in combination with trifluridine/tipiracil for pre-treated patients with unresectable, metastatic colorectal cancer with wild-type *RAS*: The phase 1/2 APOLLON study

**Target journal:** International Journal of Clinical Oncology

Takeshi Kato, Yoshinori Kagawa, Yasutoshi Kuboki, Makio Gamoh, Yoshito Komatsu, Hirofumi Yasui, Hironaga Satake, Eiji Oki, Hiroaki Tanioka, Masahito Kotaka, Akitaka Makiyama, Tadamichi Denda, Masahiro Goto, Takayuki Yoshino, Kentaro Yamazaki, Junpei Soeda, Kazunori Shibuya, Masaru Iwata, Koji Oba, Kensei Yamaguchi

Corresponding author: Dr. Kensei Yamaguchi, MD

Gastroenterological Chemotherapy Department,

Cancer Institute Hospital of Japanese Foundation for Cancer Research,

3 Chome-8-31, Ariake, Koto, Tokyo 135-8550, Japan.

**Online Resource 1. Additional information on the study methods**

***Study registration***

In addition to the study registration at the university hospital Medical Information Network Clinical Trials Registry (number UMIN000019876), the present study is registered at ClinicalTrials.gov (number NCT02613221) and Japan Pharmaceutical Information Center Clinical Trials Information (number JapicCTI-153076).

***Additional inclusion/exclusion criteria***

Patients with adequate bone marrow, hepatic, and renal functions that were determined by blood testing within the 14 days prior to enrolment were included in the study. Other exclusion criteria were receipt of radiotherapy and chemotherapy for metastatic colorectal cancer within 14 days prior to study treatment initiation (pain relief therapy for bone metastases was allowed) or receipt of investigational drugs within 28 days prior to study treatment initiation; presence or suspicion of brain metastasis; synchronous or metachronous cancers with a disease-free period of ≤5 years (excluding colorectal cancer and cured/resectable mucosal cancers); presence of effusion requiring treatment; pregnancy, lactation, or unwillingness to use appropriate contraceptive therapy; presence of any clinically serious medical condition, or disease requiring systemic steroids for treatment; serious drug hypersensitivity (excluding allergic reaction to oxaliplatin); presence of local or systemic active infection requiring treatment, or fever indicating infection; history of adverse events due to previous treatment that did not recover to Grade 1 (excluding haemoglobin levels) or Grade 2 (for peripheral neuropathy only) per the Common Terminology Criteria for Adverse Events (CTCAE; Japanese version 4.03); and other reasons based on the opinion of the investigator.

**Study procedures**

If dose-limiting toxicities (DLTs) were observed in ≤2 of the first three patients enrolled, an additional three patients were enrolled; if DLTs occurred in ≤2 of these six patients, the starting dose would be considered the recommended phase 2 dose (RP2D). If DLTs occurred in three patients at the starting dose, the dose would be de-escalated to level −1, and the 3+3 process repeated. If DLTs occurred in ≤2 of these six patients receiving the level −1 dose, this dose would be considered the RP2D. If DLTs occurred in three patients at dose level −1, the study would be discontinued.

**Online Resource 2.** Characteristics of patterns with a response, administration status of both panitumumab and FTD/TPI, and duration of progression-free survival and overall survival

*FTD/TPI* trifluridine/tipiracil, *OS* overall survival


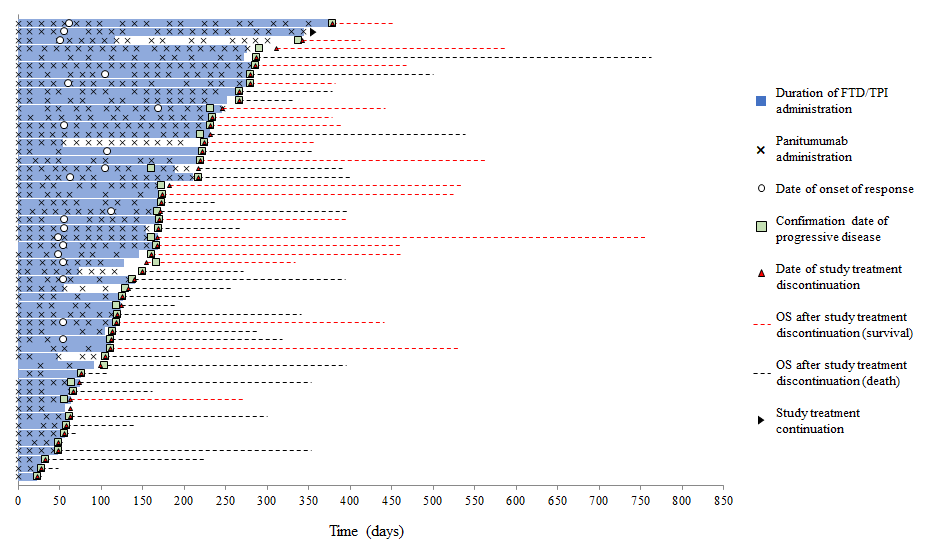


**Online Resource 3.** Subsequent treatment after the discontinuation of the study treatment

| **Subsequent chemotherapy** | **Patients (*N* = 55)** |
| --- | --- |
| Yes | 39 (70.9%) |
| Regorafenib | 25 (45.5%) |
| Others | 14 (25.5%) |
| No | 16 (29.1%) |
| Treatment continuation | 1 (1.8%) |
| Dead within 4 weeks after treatment discontinuation | 4 (7.3%) |
| BSC after treatment discontinuation | 11 (20.0%) |

Data are n (%).

*BSC* best supportive care

**Online Resource 4.** Exploratory analysis of factors affecting progression-free survival

| **Factor** |  | **Patients, *n*** | **Median progression-free survival, months**  **(95% CI)** | **Hazard ratio (95% CI)** | **p value**  **(log-rank)** |
| --- | --- | --- | --- | --- | --- |
| Age, years | <65 | 30 | 5.5  (3.79–6.21) | 0.57  (0.30–1.06) | 0.0762 |
|  | ≥65 | 24 | 7.0  (4.29–9.89) |  |  |
| Sex | Male | 28 | 6.1  (4.29–8.32) | 1.17  (0.55–2.49) | 0.6764 |
|  | Female | 26 | 5.7  (4.11–6.25) |  |  |
| ECOG performance status | 0 | 37 | 6.1  (4.29–6.57) | 0.79  (0.39–1.58) | 0.5043 |
|  | 1 | 17 | 5.8  (2.36–7.96) |  |  |
| Primary site | Right-sided | 7 | 2.9  (1.25–10.04) | 1.23  (0.45–3.36) | 0.6910 |
|  | Left-sided | 47 | 6.1  (4.46–6.57) |  |  |
| Adjuvant chemotherapy | No | 38 | 5.8  (4.11–6.50) | 1.03  (0.52–2.08) | 0.9253 |
|  | Yes | 16 | 6.2  (4.29–7.89) |  |  |
| Resection of primary tumour | No | 12 | 5.9  (1.21–8.43) | 0.74  (0.32–1.67) | 0.4611 |
|  | Yes | 42 | 5.8  (4.29–6.57) |  |  |
| Days after start of first-line therapy | <600 | 27 | 6.1  (2.29–7.82) | 0.64  (0.33–1.26) | 0.1966 |
|  | ≥600 | 26 | 5.8  (4.29–8.43) |  |  |

*CI* confidence interval, *ECOG* Eastern Cooperative Oncology Group

**Online Resource 5.** Exploratory analysis of factors affecting overall survival

| **Factor** |  | **Patients, *n*** | **Median overall** **survival, months**  **(95% CI)** | **Hazard ratio (95% CI)** | **p value**  **(log-rank)** |
| --- | --- | --- | --- | --- | --- |
| Age, years | <65 | 30 | 13.6  (9.29–NA) | 0.57  (0.26–1.27) | 0.1711 |
|  | ≥65 | 24 | 17.9  (12.18–27.29) |  |  |
| Sex | Male | 28 | 14.7  (12.18–27.29) | 1.62  (0.70–3.76) | 0.2635 |
|  | Female | 26 | 12.6  (9.68–NA) |  |  |
| ECOG performance status | 0 | 37 | 14.3  (12.21–27.29) | 1.74  (0.78–3.87) | 0.1731 |
|  | 1 | 17 | 12.6  (8.61–19.29) |  |  |
| Primary site | Right-sided | 7 | 5.1  (1.96–NA) | 0.98  (0.26–3.72) | 0.9737 |
|  | Left-sided | 47 | 14.1  (12.21–19.29) |  |  |
| Adjuvant chemotherapy | No | 38 | 14.0  (11.39–NA) | 1.38  (0.58–3.26) | 0.4669 |
|  | Yes | 16 | 14.5  (7.79–19.29) |  |  |
| Resection of primary tumour | No | 12 | 12.9  (7.11–NA) | 0.39  (0.14–1.13) | 0.0834 |
|  | Yes | 42 | 14.5  (11.39–27.29) |  |  |
| Days after start of first-line therapy | <600 | 27 | 14.1  (8.61–19.29) | 0.86  (0.39–1.88) | 0.6986 |
|  | ≥600 | 26 | 14.7  (11.39–27.29) |  |  |

*CI* confidence interval, *ECOG* Eastern Cooperative Oncology Group, *NA* not achieved

**Online Resource 6.** Exploratory analysis of factors affecting the overall response rate

| **Factor** |  | **Patients, *n*** | **Response rate, %**  **(95% CI)** | **Odds ratio (95% CI)** | **p value**  **(log-rank)** |
| --- | --- | --- | --- | --- | --- |
| Age, years | <65 | 30 | 30.0%  (14.73–49.40) | 1.78  (0.53–5.95) | 0.3479 |
|  | ≥65 | 24 | 45.8%  (25.55–67.18) |  |  |
| Sex | Male | 28 | 32.1%  (15.88–52.35) | 1.44  (0.39–5.35) | 0.5898 |
|  | Female | 26 | 42.3%  (23.35–63.08) |  |  |
| ECOG performance status | 0 | 37 | 29.7%  (15.87–46.98) | 2.36  (0.64–8.65) | 0.1963 |
|  | 1 | 17 | 52.9%  (27.81–77.02) |  |  |
| Primary site | Right-sided | 7 | 28.6%  (3.67–70.96) | 1.12  (0.14–8.95) | 0.9157 |
|  | Left-sided | 47 | 38.3%  (24.51–53.62) |  |  |
| Adjuvant chemotherapy | No | 38 | 39.5%  (24.04–56.61) | 0.45  (0.11–1.83) | 0.2671 |
|  | Yes | 16 | 31.3%  (11.02–58.66) |  |  |
| Resection of primary tumour | No | 12 | 33.3%  (9.92–65.11) | 1.30  (0.25–6.66) | 0.7529 |
|  | Yes | 42 | 38.1%  (23.57–54.36) |  |  |
| Days after start of first-line therapy | <600 | 27 | 37.0%  (19.40–57.63) | 1.05  (0.29–3.83) | 0.9451 |
|  | ≥600 | 26 | 38.5%  (20.23–59.43) |  |  |

*CI* confidence interval, *ECOG* Eastern Cooperative Oncology Group

**Online Resource 7.** Early tumour shrinkage and clinical outcomes

|  | **Full analysis set (*N* = 54)** | |  |  |
| --- | --- | --- | --- | --- |
|  | **Yes** | **No** | **Hazard ratio** | **p value** |
| Early tumour shrinkage, n (%) | 21  (38.9) | 33  (61.1) |  |  |
| Median progression-free survival, months  (95% CI) | 6.1  (5.79–8.11) | 6.3  (4.46–8.43) | 1.28  (0.68–2.41) | 0.4509 |
| Median overall survival, months  (95% CI) | 17.9  (14.29–NA) | 12.2  (9.29–14.68) | 2.67  (1.16–6.14) | 0.0206 |

*CI* confidence interval, *NA* not achieved

**Online Resource 8.** Kaplan–Meier curves of (A) progression-free survival and (B) overall survival by early tumour shrinkage

*ETS* early tumour shrinkage

**A**


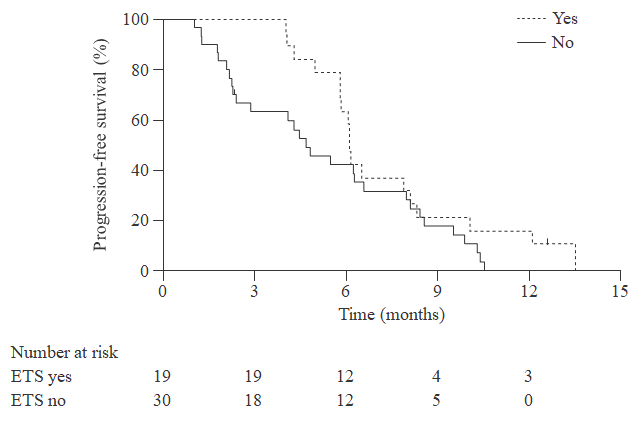


**B**


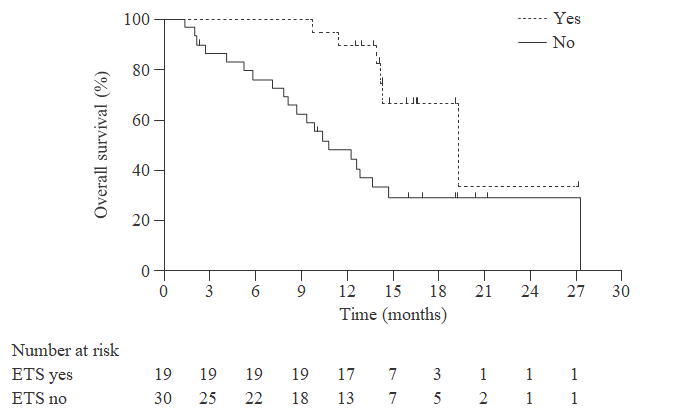


**Online Resource 9.** Kaplan–Meier curves of (A) progression-free survival and (B) overall survival by primary tumour location

**A**


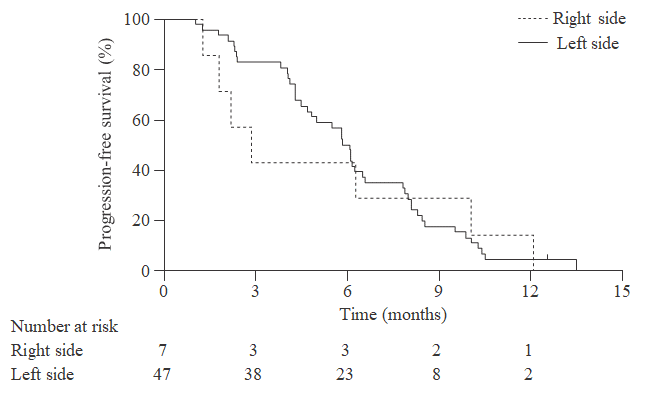


**B**


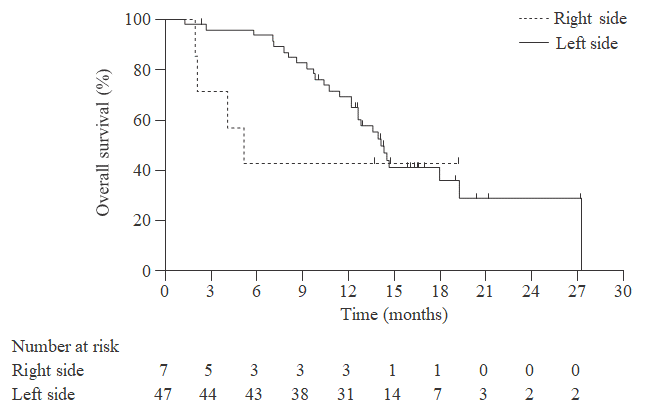


**Online Resource 10.** Change in target lesion burden over time by primary tumour location; (A) right-sided patients, (B) left-sided patients

*PD* progressive disease, *PR* partial response, *SD* stable disease.

**A**


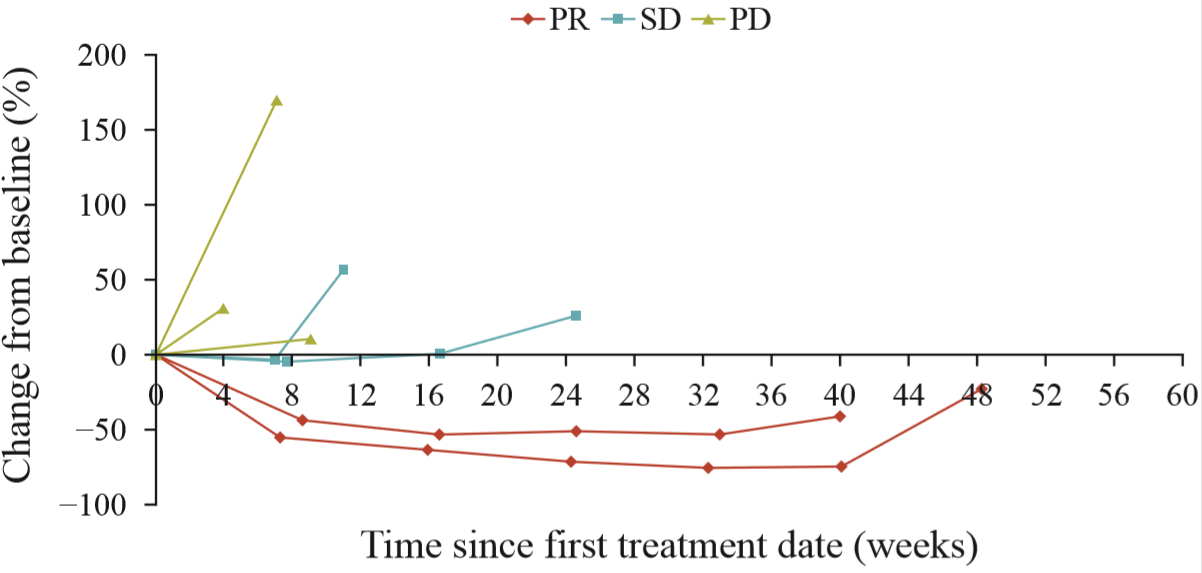


**B**

**
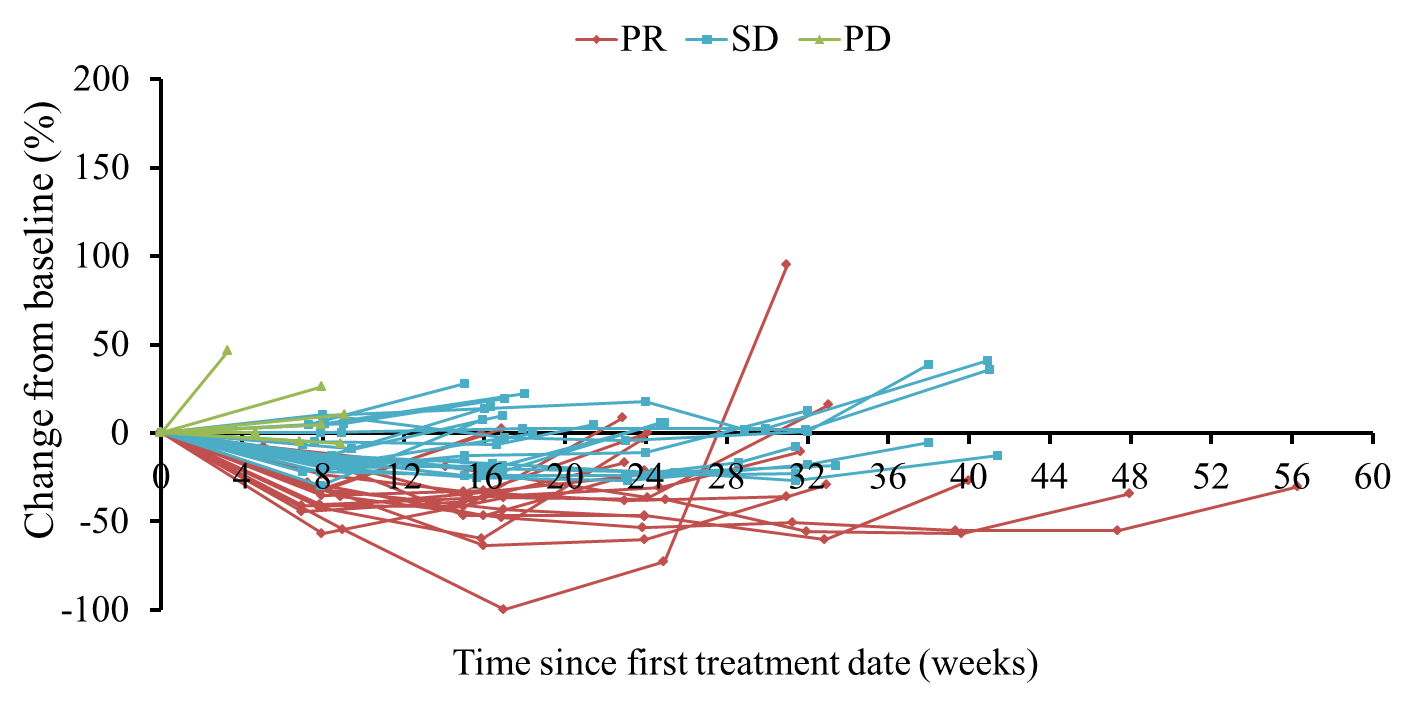
**

**Online Resource 11.** Participating sites

| Site name | Principal investigator | Number of  enrolled patients |
| --- | --- | --- |
| Kansai Rosai Hospital, Amagasaki, Japan | Yoshinori Kagawa | 9 |
| National Cancer Center Hospital East, Kashiwa, Japan | Yasutoshi Kuboki | 6 |
| Osaki Citizen Hospital, Osaki, Japan | Makio Gamoh | 5 |
| Cancer Institute Hospital of Japanese Foundation for Cancer Research, Tokyo, Japan | Kensei Yamaguchi | 4 |
| Shizuoka Cancer Center, Shizuoka, Japan | Hirofumi Yasui | 3 |
| Hokkaido University Hospital, Sapporo, Japan | Yoshito Komatsu | 3 |
| Japan Community Healthcare Organization Kyushu Hospital, Kitakyushu, Japan | Akitaka Makiyama | 2 |
| Okayama Rosai Hospital, Okayama, Japan | Hiroaki Tanioka | 2 |
| Kyushu University, Fukuoka, Japan | Eiji Oki | 2 |
| Chiba Cancer Center, Chiba, Japan | Tadamichi Denda | 2 |
| Sano Hospital, Kobe, Japan | Masahito Kotaka | 2 |
| Osaka Medical College Hospital, Takatsuki, Japan | Masahiro Goto | 2 |
| Kobe City Medical Center General Hospital, Kobe, Japan | Hironaga Satake | 2 |
| Kurume University Hospital, Kurume, Japan | Keisuke Miwa | 1 |
| Sapporo City General Hospital, Sapporo, Japan | Michio Nakamura | 1 |
| Keiyukai Sapporo Hospital, Sapporo, Japan | Hiroyuki Okuda | 1 |
| Aizawa Hospital, Matsumoto, Japan | Masato Nakamura | 1 |
| Hakodate Municipal Hospital, Hakodate, Japan | Kazuteru Hatanaka | 1 |
| Toyama University Hospital, Toyama, Japan | Takayuki Ando | 1 |
| Kitasato University Hospital, Sagamihara, Japan | Takeo Sato | 1 |
| Saitama Cancer Center, Saitama, Japan | Hiroki Hara | 1 |
| Kushiro Rosai Hospital, Kushiro, Japan | Yoshimitsu Kobayashi | 1 |
| Dongo Hospital Kenseikai, Yamatotakada, Japan | Tosio Otuji | 1 |
| Toranomon Hospital, Tokyo, Japan | Toshimi Takano | 1 |
| University of Tsukuba Hospital, Tsukuba, Japan | Toshikazu Moriwaki | 1 |
